# Screening, Identification, and Fermentation of *Brevibacillus laterosporus* YS-13 and Its Impact on Spring Wheat Growth

**DOI:** 10.3390/microorganisms13061244

**Published:** 2025-05-28

**Authors:** Wenjing Zhang, Xingxin Sun, Zele Wang, Jiayao Li, Yuanzhe Zhang, Wei Zhang, Jun Zhang, Xianghan Cheng, Peng Song

**Affiliations:** 1 College of Agriculture, Henan University of Science and Technology, Luoyang 471000, China; zwj120034@163.com (W.Z.); sunxingxin1015@163.com (X.S.); 13083798245@163.com (Z.W.); 17337216330@163.com (J.L.); 19837936221@163.com (Y.Z.); zhangjun0105@126.com (J.Z.); chengxianghan@haust.edu.cn (X.C.); 2 Luoyang Agricultural Technology Promotion Service Center, Luoyang 471000, China; 13598180468@163.com

**Keywords:** phosphate-solubilizing bacteria, phosphate-solubilizing ability, identification, response surface methodology, spring wheat

## Abstract

The low availability of phosphorus (P) in soil has become a critical factor limiting crop growth and agricultural productivity. This study aimed to isolate and evaluate a bacterial strain with high phosphate-solubilizing capacity to improve soil phosphorus utilization and promote crop growth. A phosphate-solubilizing bacterium, designated as YS-13, was isolated from farmland soil in Henan Province, China, and identified as *Brevibacillus laterosporus* based on morphological characteristics, physiological and biochemical traits, and 16S rDNA sequence analysis. Qualitative assessment using plate assays showed that strain YS-13 formed a prominent phosphate solubilization zone on organic and inorganic phosphorus media containing lecithin and calcium phosphate, with D/d ratios of 2.28 and 1.57, respectively. Quantitative evaluation using the molybdenum–antimony colorimetric method revealed soluble phosphorus concentrations of 21.24, 6.67, 11.73, and 17.05 mg·L^−1^ when lecithin, ferric phosphate, calcium phosphate, and calcium phytate were used as phosphorus sources, respectively. The fermentation conditions for YS-13 were optimized through single-factor experiments combined with response surface methodology, using viable cell count as the response variable. The optimal conditions were determined as 34 °C, 8% inoculum volume, initial pH of 7.55, 48 h incubation, 5 g L^−1^ NaCl, 8.96 g L^−1^ glucose, and 8.86 g L^−1^ peptone, under which the viable cell count reached 6.29 × 10^8^ CFU mL^−1^, consistent with the predicted value (98.33%, *p* < 0.05). The plant growth-promoting effect of YS-13 was further validated through a pot experiment using *Triticum aestivum* cv. Jinchun 6. Growth parameters, including plant height, fresh biomass, root length, root surface area, root volume, and phosphorus content in roots and stems, were measured. The results demonstrated that YS-13 significantly enhanced wheat growth, with a positive correlation between bacterial concentration and growth indicators, although the growth-promoting effect plateaued at higher concentrations. This study successfully identified a high-efficiency phosphate-solubilizing strain, YS-13, and established optimal culture conditions and bioassay validation, laying a foundation for its potential application as a microbial inoculant and providing theoretical and technical support for reducing phosphorus fertilizer inputs and advancing sustainable agriculture.

## 1. Introduction

Phosphorus (P) is a vital nutrient for plant growth and development, playing a key role in processes such as cell division, energy transfer, signal transduction, nucleic acid synthesis, and photosynthesis [1,2]. Research by Nayak et al. highlights that, during most stages of plant growth, the relative importance of nutrients follows this order: nitrogen > phosphorus > potassium > zinc [3]. Plants primarily obtain phosphorus from minerals and organic compounds in the soil. However, compared to other nutrients, the availability of phosphorus in soil is relatively low, ranging from 400 to 1000 mg kg^−1^ [4,5]. When the amount of directly available phosphorus is less than 0.2% of total phosphorus, plants show symptoms of phosphorus deficiency, including slow growth, leaf chlorosis, blackening, and reduced fruiting [6]. The impact of phosphorus deficiency on plant growth is similar to nitrogen deficiency [7]. In most soils, only a small fraction—around 0.1%—of total phosphorus is available to plants. To meet phosphorus demands, farmers typically rely on fertilizers [8]. However, the application of phosphorus fertilizers can vary widely based on soil type, with different dosages and timing depending on the use of rock phosphate, phosphorite, or chemical fertilizers [9]. While plants absorb a portion of the phosphorus applied to the soil, the rest often reacts with calcium (Ca^2+^), magnesium (Mg^2+^), iron (Fe^3+^), or aluminum (Al^3+^) ions to form insoluble phosphates [10]. As a result, the efficiency of phosphorus fertilizer use is typically low, with only about 25% of applied phosphorus being effectively utilized by plants, whether in the form of single superphosphate or diammonium phosphate [11,12]. Excessive accumulation of insoluble phosphates in the soil can disrupt beneficial rhizosphere microbes, leading to microbial imbalances. This not only reduces soil fertility but also results in lower crop yields [13].

Soil is home to a wide variety of phosphate-solubilizing bacteria (PSB) capable of converting insoluble phosphates, such as phosphate rock powder, into soluble forms. This process increases the available phosphorus in the soil, enhancing plant phosphorus nutrition and improving crop yields [14]. While PSB are the most abundant and diverse microorganisms involved in phosphate solubilization, fungi and actinobacteria also contribute to this process. Phosphate-solubilizing fungi, however, are less prevalent and are primarily found in genera such as *Penicillium*, *Aspergillus*, *Fusarium*, and *Rhizoctonia* [15]. Microbial phosphate solubilization is often associated with the secretion of organic acids, though other mechanisms may also play a role in this process [16]. The screening of PSB species and research into their effectiveness have been widely explored. Furthermore, phosphate-solubilizing fungi have been shown to significantly benefit the growth of various plants, including sunflower and tobacco [17,18].

Despite the growing interest in phosphate solubilization, no studies have yet focused on optimizing the conditions for microbial phosphate solubilization. This study aimed to enhance soil phosphorus availability by adhering to the principles of bioremediation, focusing on screening a bacterium with high phosphorus-solubilizing ability. By optimizing its fermentation conditions and validating its growth-promoting effects, a solid foundation for its formulation application was established. Furthermore, this study provides theoretical and technical support for reducing phosphorus fertilizer usage and promoting sustainable agricultural development.

## 2. Materials and Methods

### 2.1. Characteristics and Identification of the Strain

Soil samples were collected from farmland in Luoyang City (Henan University of Science and Technology), as well as from Xinyang and Sanmenxia Cities in Henan Province. These samples were cultured on LB agar medium (composition: 10 g tryptone, 5 g yeast extract, 10 g NaCl, 15 g agar) for further analysis [19]. Strain characteristics were determined following the method outlined by Deng Meikui [20]. The strain was then inoculated at a single point onto LB agar and incubated at 30 °C for 24 h to observe colony morphology.

The growth characteristics of strain YS-13 and Gram staining observation were conducted using an optical electron microscope (EX30 model; Hunan Honglin Scientific Instrument Co., Ltd., Hunan, China). Morphological characteristics were examined using a scanning electron microscope (JSM-IT200 model; Beijing Scientific Instrument Co., Ltd., Beijing, China). The strain was subjected to the following physiological and biochemical tests: catalase reaction, strict aerobiosis, methyl red test, Voges–Proskauer (V-P) test, nitrate reduction, indole production, citrate utilization, starch hydrolysis, casein hydrolysis, hydrogen sulfide production, sugar alcohol fermentation, salt tolerance tests with 2%, 5%, and 7% (*w*/*v*) NaCl concentrations, and heat tolerance tests at 45 °C and 65 °C [21,22].

Colony morphology and staining characteristics were recorded using the methods described by Liu Xingang [23]. The genomic DNA of the purified strain was extracted with a bacterial genomic DNA extraction kit. PCR amplification of the 16S rDNA was carried out using universal bacterial primers 27F and 1492R. The PCR reaction mix (25 μL) included 2.5 μL of 10× buffer, 0.5 μL of Taq enzyme, 0.5 μL of primer 27F, 0.5 μL of primer 1492R, 1 μL of DNA template, and 20 μL of ddH_2_O. The PCR reaction procedure: pre-denaturation at 94 °C for 5 min; denatured for 45 s at 94 °C, annealed for 45 s at 55 °C, extended for 1 min at 72 °C (30 cycles); and 72 °C extension for 10 min [24].

The PCR products were sent to Sangon Biotech Co., Ltd. (Shanghai, China) for sequencing. BLAST (Version 2.15.0; NCBI, Bethesda, Montgomery County, MD, USA) results were downloaded, and phylogenetic trees were constructed using MEGA software (Version 7.0.26; Mega Limited, New Zealand). The resulting 16S rDNA sequences were compared with those in the NCBI database, BLAST results were downloaded, and phylogenetic trees were constructed using MEGA software (Version 7.0.26; Mega Limited, Auckland, New Zealand).

### 2.2. Evaluation of Phosphate-Solubilizing Capacity

Single colonies were selected and inoculated onto Mongina organic phosphate solid medium (lecithin) and Mongina inorganic phosphate solid medium (calcium phosphate) plates [25]. The plates were incubated at 30 °C in an inverted position for 1–2 days. Colonies showing distinct morphological differences and visible phosphate-solubilizing zones (based on size, surface structure, texture, glossiness, and color) were selected for further analysis. These colonies were then purified by repeated streaking on fresh plates. The purified strains were numbered and stored for future use.

The purified strain was inoculated into LB liquid medium and cultured on a shaker (SKY-2102 model; Changzhou Jintan Jingda Instrument Manufacturing Co., Ltd., China) at 30 °C and 120 r min^−1^, with uninoculated medium incubated under identical conditions at 30 °C and 120 r min^−1^ for 24 h. The bacterial suspension was then adjusted to a concentration of 1 × 10^8^ CFU mL^−1^ to prepare the seed inoculum [26]. This inoculum was added to 500 mL Erlenmeyer flasks containing 150 mL of Mongina organic phosphate liquid medium (lecithin) at a 2% inoculation volume. The cultures were incubated on a shaker at 30 °C and 120 r min^−1^, with an uninoculated medium as the control. Each treatment was conducted in triplicate, and samples were collected every 24 h for up to 5 days.

A phosphate standard curve was constructed [27], and the available phosphorus content in the samples was measured using the molybdenum–antimony colorimetric method [28]. The phosphate-solubilizing ability of the strain was then calculated by subtracting the blank control values.

### 2.3. Phosphate-Solubilizing Characteristics of the Strain Across Different Phosphorus Sources

Mongina solid media containing four different phosphorus sources—calcium phosphate, iron phosphate, calcium phytate, and lecithin—were prepared. The purified strain YS-13 was inoculated onto each of the four media plates, with five inoculation points per plate. The plates were incubated at 30 °C for 7 days to observe strain growth and the formation of phosphate-solubilizing zones. A cross-measurement method was used to measure the colony diameter (d, mm) and the phosphate-solubilizing zone diameter (D, mm). The D/d ratio was calculated to assess the phosphate-solubilizing ability of the strain YS-13 with different phosphorus sources.

The purified strain was inoculated into LB liquid medium and cultured at 30 °C with shaking at 120 r min^−1^ for 1 day. The bacterial suspension was adjusted to a concentration of 1 × 10^8^ CFU mL^−1^ to prepare the seed inoculum. This inoculum was then added to Mongina liquid media containing different phosphorus sources—calcium phosphate, iron phosphate, calcium phytate, and lecithin—at a 2% inoculum volume. The cultures were incubated at 30 °C with shaking at 120 r min^−1^ for 7 days. The available phosphorus content in the samples was measured using the molybdenum–antimony colorimetric method. The phosphate-solubilizing ability of the strain was calculated by subtracting the control values, and the available phosphorus in the fermentation supernatant was determined using a phosphate standard curve.

### 2.4. Experimental Design and Process Optimization

In the fermentation test, the number of live bacteria was used as the determination index. For each treatment, three repetitions were performed, and the average value was calculated. The number of live bacteria in the fermentation solution was determined by the plate counting method [29]. Single-Factor Experiments [30,31]: Using the previously screened strain as the test strain, the medium composition and fermentation conditions were optimized. The viable cell count was measured using the plate counting method. Based on the basic medium composition and fermentation conditions, the effects of various factors were studied, including carbon sources (glucose, sucrose, soluble starch, fructose, and lactose), nitrogen sources (ammonium sulfate, beef extract, peptone, and ammonium nitrate), and inorganic salts (calcium chloride, magnesium chloride, magnesium sulfate, and potassium chloride).

The optimal carbon source, nitrogen source, and inorganic salt were identified, and the best medium composition was determined. Furthermore, the effects of fermentation temperature (24–38 °C), initial pH (5–9), amount of inoculum (2.0%-12.0%), cultivation time (24–60 h), and rotational speed (120–200 r min^−1^) on the viable cell count of the strain were evaluated to establish the optimal fermentation conditions.

The plate counting method [32] was used to determine the viable cell count. Three 10-fold serial dilutions of the seed culture were prepared. From each dilution, 1 mL was transferred to sterilized Petri dishes and mixed with nutrient agar. After incubation at the specified temperature for 48 h, the colonies were counted. The total number of bacterial colonies per mL of fermentation broth was then calculated based on the dilution factor.

Plackett–Burman Experiment: Design-Expert 13 software was used for the Plackett–Burman trial design. Seven key medium components influencing the viable cell count of the strain were selected as independent variables, with the viable cell count in the fermentation broth as the response variable. A Plackett–Burman design with *n* = 12 was employed, where each factor was tested at two levels, high (+1) and low (−1), to identify the significant factors affecting the cell count.

Steepest Ascent Experiment and Box–Behnken Experiment: The coefficient values of significant factors identified in the Plackett–Burman experiment were used to determine the step size and direction for the steepest ascent experiment. Factors with positive coefficients were assigned to the high level, while those with negative coefficients were assigned to the low level. The highest viable cell count obtained from the steepest ascent experiment was then used as the starting point for the Box–Behnken design.

Using the significant factors identified in the Plackett–Burman experiment as independent variables and the viable cell count of the strain in the fermentation broth as the response variable, a multi-factor, multi-level response surface experiment was conducted. A quadratic regression model was established to describe the relationship between the independent variables and the response. The optimal fermentation conditions for maximizing the strain’s viable cell count were determined.

### 2.5. Design of the Pot Experiment

Pour the wheat seeds into a small beaker under the laminar flow hood and add 10 mL of 10% ethanol to soak for 3 min. Then, discard the ethanol (including the floating seeds) and rinse once with sterile water. Next, add 1% sodium hypochlorite and soak for 5–10 min. Finally, rinse 3–4 times with sterile water.

The strain inoculant was prepared as follows: The strain was cultured in LB medium, and a bacterial suspension was prepared by culturing it in a shaker at 30 °C for 18 h. The bacterial suspension was centrifuged at 4000 r min^−1^ for 10 min. The bacterial pellet was rinsed with sterile water, collected, and diluted with sterile water to prepare the stock solution (1 × 10^8^ CFU mL^−1^) for further use in the experiment. The stock solution was diluted with sterile water to obtain concentrations of the strain inoculant of 10^6^ CFU mL^−1^, 10^7^ CFU mL^−1^, and 10^8^ CFU mL^−1^ [33].

Experimental soil: The soil was collected from the 0–20 cm layer of the farm at Henan University of Science and Technology. The alkali-hydrolyzable nitrogen, available phosphorus, available potassium, and organic matter content of the soil were 33.86 mg kg^−1^, 8.46 mg kg^−1^, 118.2 mg kg^−1^, and 10.72 g kg^−1^, respectively. The soil pH was 7.35.

The pot experiment involved four treatments: a control group with original soil (CK), original soil with a low concentration (10^6^ CFU mL^−1^) of the strain inoculant (TL), original soil with a medium concentration (10^7^ CFU mL^−1^) of the strain inoculant (TM), and original soil with a high concentration (10^8^ CFU mL^−1^) of the strain inoculant (TH) [34]. Soil samples were prepared and equilibrated at room temperature for 7 days prior to the experiment. Each pot contained 5 kg of mixed soil, with six replicates for each treatment. Wheat variety Jinchun No. 6 was sown evenly in each pot. The treated groups received 400 mL of strain inoculant every 14 days, while the control group received 400 mL of liquid medium without the strain inoculant. Additionally, each pot was watered with 200 mL of water every 4 days. Wheat growth was monitored, and samples were collected every 7 days. A total of four samplings were conducted over a period of 28 days. Three replicates were taken from each pot to measure the growth indicators [35,36].

### 2.6. Growth Index Determination

After a random selection of three wheat plants from each pot, their plant height was measured with a ruler and the average value was calculated; the wheat roots were washed thoroughly and then blotted with paper to remove excess moisture, and the fresh weights of the roots and shoots were measured separately using a balance.

The phosphorus content in the wheat roots and shoots was determined by the wet digestion method. Weigh 0.15 g of ground, dried wheat sample, add concentrated sulfuric acid and a small amount of deionized water to moisten the sample, and heat it at a low temperature. Once white smoke appears and the mixture turns brownish-black, gradually add 30% H_2_O_2_ for oxidative digestion until the solution becomes clear or colorless, then make up the volume to 50 mL and let it sit overnight. For the phosphorus content measurement, take 2.5 mL of the supernatant, dilute it to 30 mL, add 2 drops of 2,6-dinitrophenol indicator, adjust the pH to yellow using 6 mol L^−1^ NaOH, and then add sulfuric acid to bleach the solution. Next, add 5 mL of molybdenum–antimony reagent, dilute to 50 mL, and allow the color to develop for 30 min. Measure the absorbance at 720 nm using a colorimeter, calibrating to zero with a blank sample, and calculate the phosphorus content based on the calibration curve [37].

Phosphorus content was calculated using the following formula:

P (μg/g) = *β* × V/m × V_2_/V_1_ (1)

where P represents the phosphorus content in the wheat roots and shoots (μg g^−1^), *β* represents the phosphorus concentration in the test solution (μg mL^−1^), V represents the volume of the sample solution (mL), m represents the mass of the sample (g), V_1_ represents the sampling volume (mL), and V_2_ represents the color development volume (mL).

To analyze the wheat root morphology, including total root length, root surface area, root volume, and average diameter, first collect and clean the wheat roots, removing soil impurities and soaking them in deionized water. Lay the cleaned roots flat within the scanning area of a root scanner, ensuring that the roots do not overlap. Scan at an appropriate resolution (400 dpi) to capture the root images. After scanning, import the images into the RootSnap software (Version 1.2.5; PlantVillage, Pennsylvania, PA, USA), which automatically recognizes the roots and extracts relevant data such as total root length, average diameter, root surface area, and total volume.

### 2.7. Statistical Analyses

All experimental data underwent an analysis of variance (ANOVA) using the SPSS Statistics software (SPSS 22.0; International Business Machines Corporation, Armonk, NY, USA). The mean values for each treatment were compared using Duncan’s multiple range test to determine significant differences (at the 0.05 level). Data were presented as means ± standard error. Response surface analysis was performed with Design-Expert (Version 13; Stat-Ease, Minneapolis, MN, USA), while phylogenetic tree construction was conducted using MEGA 11.0 software (Version 7.0.26; Mega Limited, Auckland, New Zealand). Graphical representations were generated with Origin software (Origin 2021; OriginLab Corporation, Northampton, MA, USA).

Design-Expert 13 software was used to analyze the experimental data, and the analysis of variance and regression equation were tested by F-test. *p* < 0.05 meant that the difference was significant. *R*^2^ was used to represent the fitting of multiple regression models, and *R*^2^ > 0.9 was judged to be optimal. *p* > 0.05 meant that the lack of fit was not significant, indicating that the regression model fits the data well. To validate the predicted values obtained using the response surface method, fermentation experiments were carried out under optimized conditions. Each trial was repeated three times, and the average value was calculated. By comparing the trial results with the predicted values, the reliability, accuracy, and practicality of the fermentation conditions optimized using the response surface method were determined [38].

## 3. Results

### 3.1. Strain Identification and Phosphate-Solubilizing Capacity

#### 3.1.1. Isolation and Screening of Phosphate-Solubilizing Strains

A total of 54 bacterial strains, 7 actinobacteria strains, and 3 fungal strains were isolated from the soil samples. Screening using Mongina organic and inorganic phosphate solid media revealed 28 bacterial strains with phosphate-solubilizing activity, 16 of which showed more significant effects. These strains were numbered YS-1 to YS-16. Among them, the strain YS-13 exhibited the largest phosphate-solubilizing zone and was selected for further identification and analysis.

#### 3.1.2. Phosphate-Solubilizing Capacity of the Strain YS-13

**Figure 1:**
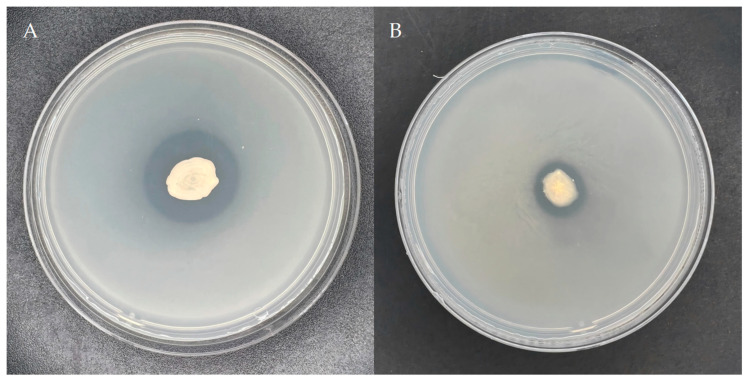
Plate phosphorus solubilization effect of the strain YS-13. (**A**) Illustrates the phosphate-solubilizing effect of the strain on NBRIP organic phosphorus (lecithin) and (**B**) inorganic phosphorus (calcium phosphate) media plates.

Following 5 days of cultivation on Mongina organic phosphate (lecithin) solid medium, a distinct phosphorolytic halo was observed surrounding the colonies of strain YS-13, as shown in Figure 1. Quantitative analysis revealed a phosphate-solubilizing zone diameter (D) of 25.73 mm and a colony diameter (d) of 11.28 mm, yielding a D/d ratio of 2.28. These results demonstrate significant organic phosphate-solubilizing capacity in strain YS-13, consistent with high-efficiency phosphate metabolism phenotypes.

Similarly, following 5-day incubation of the strain YS-13 onto Mongina inorganic phosphate (calcium phosphate) medium after bacterial inoculation, a clear inorganic phosphate-solubilizing zone appeared around the colony, as shown in Figure 1. The diameter of the phosphate-solubilizing zone (D) was 11.94 mm, and the colony diameter (d) was 7.60 mm, resulting in a D/d ratio of 1.57. These results indicate that the strain YS-13 also exhibits good inorganic phosphate-solubilizing ability.

#### 3.1.3. Phosphate-Solubilizing Capacity of the Strain YS-13 with Various Phosphorus Sources

As shown in Table 1, strain YS-13 demonstrated varying phosphate-solubilizing capacities across different phosphorus sources, with the phosphate-solubilizing zone diameter to colony diameter ratio (D/d) following this order: iron phosphate (1.20) < calcium phosphate (1.63) < calcium phytate (2.09) < lecithin (2.34), reflecting superior activity on organic phosphorus, particularly lecithin. Statistical analysis revealed significant differences (*p* < 0.05) in D/d ratios across phosphorus sources, with the highest value observed for lecithin. In liquid media, the available phosphorus concentrations followed a similar trend: lecithin (21.24 mg L^−1^) > calcium phytate (17.05 mg L^−1^) > calcium phosphate (11.73 mg L^−1^) > iron phosphate (6.67 mg L^−1^). The variability in phosphorus concentrations was also statistically significant (*p* < 0.05), reinforcing the superior solubilizing efficiency of YS-13 on organic phosphorus sources. The results were summarized in Table 1, where the means and standard deviations of phosphate-solubilizing efficiency were presented alongside the statistical significance of the differences between treatments [39,40].

#### 3.1.4. Morphological Characterization of the Strain YS-13

The colony and cell morphology of the strain YS-13 were examined. On Mongina organic phosphate medium, the strain YS-13 formed yellow-brown, circular or oval colonies with smooth, neat edges, a raised center, and a translucent texture, as detailed in Table 2. The bacterial cells were short, thick rods with blunt, rounded ends, measuring 0.5–0.9 μm by 2.0–3.0 μm, as detailed in Table 2. The spores were elliptical and located either centrally or subterminally. Additionally, the strain had peritrichous flagella and a capsule.

**Figure 2:**
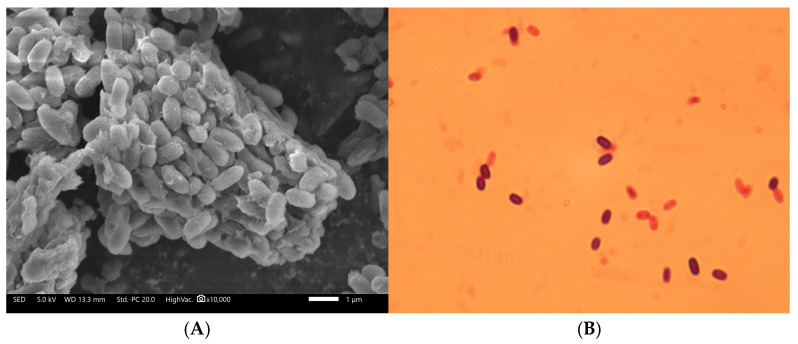
Microbial morphology of the strain YS-13. (**A**) Electron micrograph of the strain YS-13 at 10,000× magnification. (**B**) Gram staining image of the strain YS-13 at 1000× magnification.

The Gram staining results varied depending on the growth phase. During the logarithmic phase, the cells stained Gram-positive, while in the late stationary and death phases, they stained Gram-negative, as shown in Figure 2.

#### 3.1.5. Physiological and Biochemical Profiling of the Strain YS-13

The physiological and biochemical characteristics of strain YS-13 were summarized in Table 3.

#### 3.1.6. Molecular Characterization of the Strain YS-13

**Figure 3:**
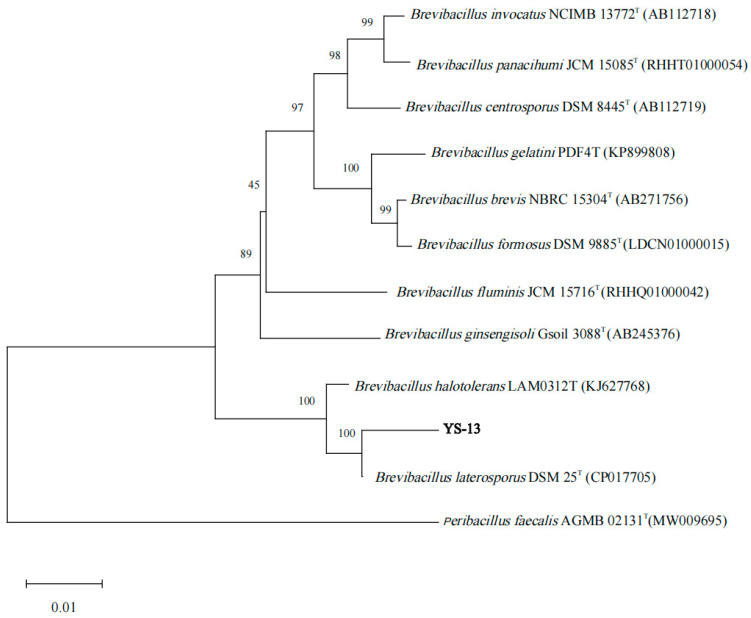
Phylogenetic tree was constructed on the basis of the 16S rDNA sequence in YS-13 and homologous sequences obtained from the NCBI database. MEGA software was used to construct the phylogenetic tree according to the neighbor-joining method, with 1000 bootstrap replicates. Only nodes with bootstrap values greater than 50% were displayed. The superscripted “T” indicates type strain.

The bacterial genome was utilized as the template for PCR amplification. The resulting product was analyzed by 1.5% agarose gel electrophoresis, revealing a fluorescent band at approximately 1400 bp. Sequencing of the amplified product confirmed a 16S rDNA fragment spanning 1381 bp. A phylogenetic tree was constructed using MEGA 11.0 software (Figure 3), demonstrating that strain YS-13 exhibited the closest evolutionary relationship to *B. laterosporus* DSM 25^T^ (GenBank accession number: CP017705), with 99.06% sequence homology. Combined morphological observations, physiological–biochemical profiling, and 16S rDNA sequence analysis supported the taxonomic identification of strain YS-13 as *B. laterosporus*. The strain was deposited at the General Microbiology Center of the China Committee for Culture Collection of Microorganisms (CGMCC, Beijing, China) on 17 July 2023, under accession number CGMCC No. 27938, and its genomic and phenotypic data have been publicly archived in the CGMCC repository.

### 3.2. Optimization of Fermentation Conditions

**Figure 4:**
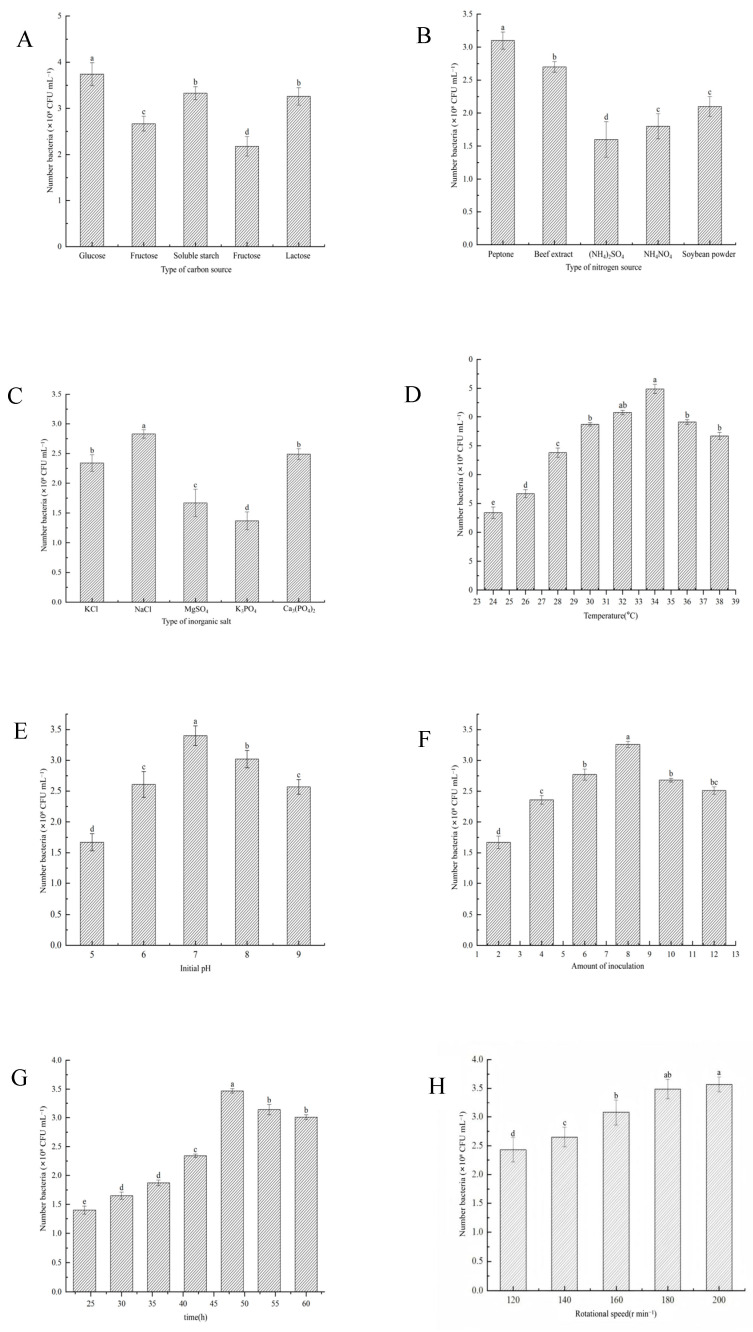
Effects of medium components and culture conditions on the growth of the strain YS-13. (**A**) Type of carbon sources. (**B**) Type of nitrogen sources. (**C**) Type of inorganic salts. (**D**) Temperature. (**E**) Initial pH. (**F**) Amount of inoculum. (**G**) Time. (**H**) Rotational speed. Note: Different letters above the barsindicate significant differences (*p* < 0.05).

As shown in Figure 4, fermentation optimization experiments were carried out to identify the best medium components and culture conditions for maximizing the viable cell count of the strain YS-13. Factors such as carbon source, nitrogen source, inorganic salts, temperature, amount of inoculum, pH, and cultivation time were assessed. The results showed that glucose was the most effective carbon source, the best nitrogen source was ammonium sulfate, and NaCl was the most efficient inorganic salt. The optimal fermentation conditions were determined to be a temperature of 34 °C, an amount of inoculum of 8.0%, a pH of 7, and a rotational speed of 180 r min^−1^. Under these conditions, the viable cell count reached its peak after 48 h of cultivation. These findings lay the groundwork for further optimization of large-scale fermentation processes for *B. laterosporus* YS-13.

Based on the basic nutritional requirements for microbial growth and the results of preliminary single-factor experiments, eight factors were selected for further analysis: glucose (*X*_1_), peptone (*X*_2_), sodium chloride (*X*_3_), temperature (*X*_4_), initial pH (*X*_5_), amount of inoculum (*X*_6_), rotational speed (*X*_7_), and time (*X*_8_). Each factor was tested at two levels, represented as −1 (low level) and +1 (high level). A total of 12 experiments were performed, with the factors and their respective levels shown in Table 4.

As shown in Table 5 and in Table A1 of Appendix A, glucose (*X*_1_), peptone (*X*_2_), and initial pH (*X*_5_) were identified as the key factors influencing the model (*p* < 0.05). These variables were, therefore, selected for further optimization experiments.

To identify the optimal conditions for glucose (*X*_1_), peptone (*X*_2_), and initial pH (*X*_5_), a steepest ascent experimental design was conducted based on the parameters listed in Table 5. The experimental results were shown in Table 6. Under condition no. 4, the viable cell count of *B. laterosporus* reached its peak at 6.11 × 10^8^ CFU mL^−1^, making this condition the chosen central point for subsequent experiments.

To investigate the interactions among glucose (*X*_1_), peptone (*X*_2_), and initial pH (*X*_5_), a three-factor, three-level response surface experiment was conducted. The experimental design and results were presented in Table 7.

The data presented in Table 7 were analyzed using Design-Expert 13 software to conduct a response surface regression analysis. A quadratic regression model was developed to describe the relationship between the viable cell count of *B. laterosporus* and the three factors: glucose (*X*_1_), peptone (*X*_2_), and initial pH (*X*_5_). The resulting polynomial regression equation was as follows: Viable cell count = −138.811 + 3.83217 *X*_1_ + 4.30411 *X*_2_ + 28.8625 *X*_5_ − 0.0788889 *X*_1_*X*_2_ − 0.06 *X*_1_*X*_5_ − 0.05 *X*_2_*X*_5_ − 0.149556 *X*_1_^2^ − 0.181778 *X*_2_^2^ − 1.846 *X*_5_^2^.

The quadratic model and the results of the variance analysis were shown in Table A2 of Appendix A. The model had a *p*-value of 0.0001, indicating high statistical significance, while the lack-of-fit *p*-value was 0.6904, suggesting that the lack of fit was not significant. The determination coefficients (*R*^2^ = 0.9947, *R*^2^_Adj_ = 0.9878, and *R*^2^_Pre_ = 0.9700) showed a difference of less than 0.2, confirming that the model provided a good fit and had strong predictive capability. The Box–Behnken design demonstrated high reliability and accuracy. Additionally, the effects of *X*_1_, *X*_2_, *X*_5_, *X*_1_*X*_2_, *X*_1_^2^, *X*_2_^2^, and *X*_5_^2^ were all highly significant (*p* < 0.01).

**Figure 5:**
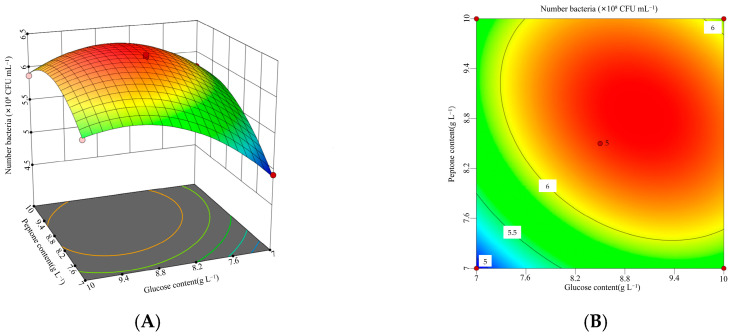
Interaction between glucose and peptone concentrations and corresponding response surface plot. (**A**) Interaction plot for the glucose concentrations and peptone concentrations. (**B**) Three-dimensional interaction plot for the glucose concentrations and peptone concentrations.

**Figure 6:**
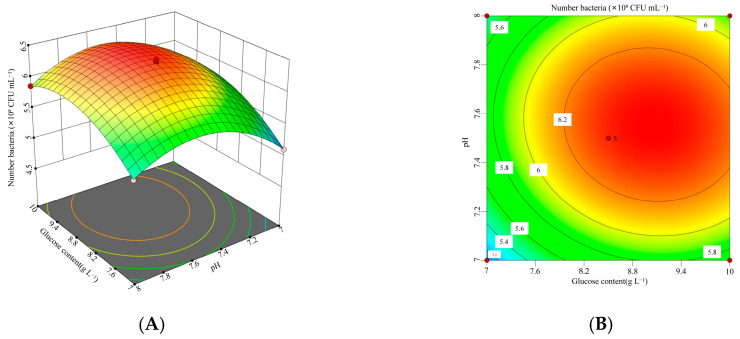
Interaction between glucose concentration and initial pH and corresponding response surface plot. (**A**) Interaction plot for the glucose concentrations and initial pH. (**B**) Three-dimensional interaction plot for the glucose concentrations and initial pH.

**Figure 7:**
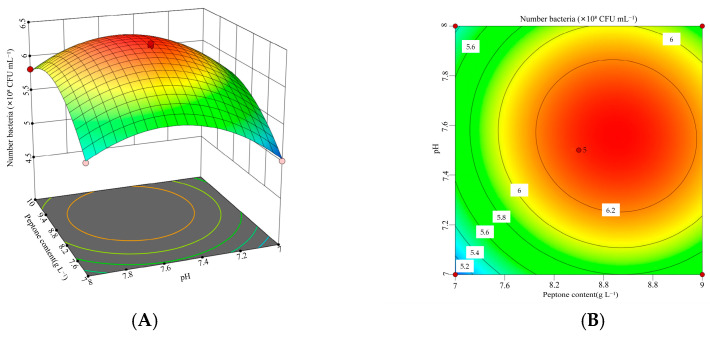
Interaction between peptone concentration and initial pH and corresponding response surface plot. (**A**) Interaction plot for the peptone concentrations and initial pH. (**B**) Three-dimensional interaction plot for the peptone concentrations and initial pH.

According to the response surface analysis shown in Figure 5, Figure 6 and Figure 7, the viable cell count initially increased and then decreased as the values of two interacting factors increased. This indicates that each pair of interacting factors has an optimal combination for maximizing the response. Using Design-Expert 13 software, the optimal levels of the significant factors were determined to be 8.96 g L^−1^ for glucose, 8.86 g L^−1^ for peptone, and 7.55 for initial pH. Under these optimized conditions, the model predicted the maximum viable cell count to be 6.40 × 10^8^ CFU mL^−1^. To validate the model, three independent experiments were performed under these conditions, yielding an average viable cell count of 6.29 × 10^8^ CFU mL^−1^, which was 98.33% of the predicted value (*p* < 0.05). These results demonstrate that the model accurately predicted the viable cell count.

### 3.3. Impact of B. laterosporus YS-13 Inoculant on Wheat Growth

Wheat is highly sensitive to phosphorus availability. Phosphorus deficiency in wheat slows growth, stunts plant development, and restricts root formation, which reduces the number of secondary roots and impairs stem and leaf growth [41]. To assess these effects, measurements were taken of wheat plant height, fresh shoot weight, root length, fresh root weight, and phosphorus content in both roots and stems.

#### 3.3.1. Effect of *B. laterosporus* YS-13 at Different Concentrations on Plant Height

**Figure 8:**
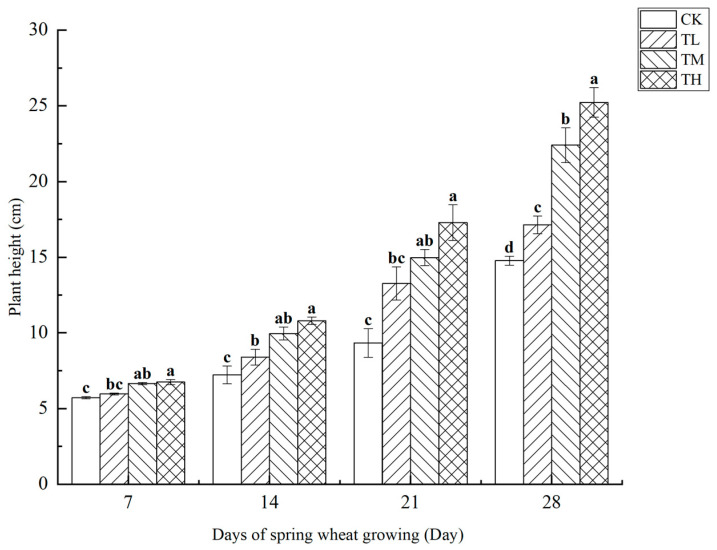
Changes in wheat plant height at different stages under inoculation with different concentrations of the *B. laterosporus* YS-13 inoculant. Note: Different letters above the barsindicate significant differences (*p* < 0.05).

As shown in Figure 8, treatments with varying concentrations of *B. laterosporus* significantly enhanced wheat plant height (*p* < 0.05). Over the first 14 days of growth, the plant height in the TH and TM groups was notably higher than in the TL group, which also showed a significant difference compared to CK. However, plant height did not show a direct correlation with bacterial concentration. On days 21 and 28, significant differences in plant height were observed across all treatment groups (*p* < 0.05), with the TH group showing the greatest increase. By day 28, the plant heights in the TH, TM, and TL groups were 70.75%, 51.73%, and 29.59% higher, respectively, compared to the control (*p* < 0.05). The most substantial growth occurred between days 21 and 28.

#### 3.3.2. Effect of *B. laterosporus* YS-13 at Different Concentrations on the Fresh Weight of Plants

**Figure 9:**
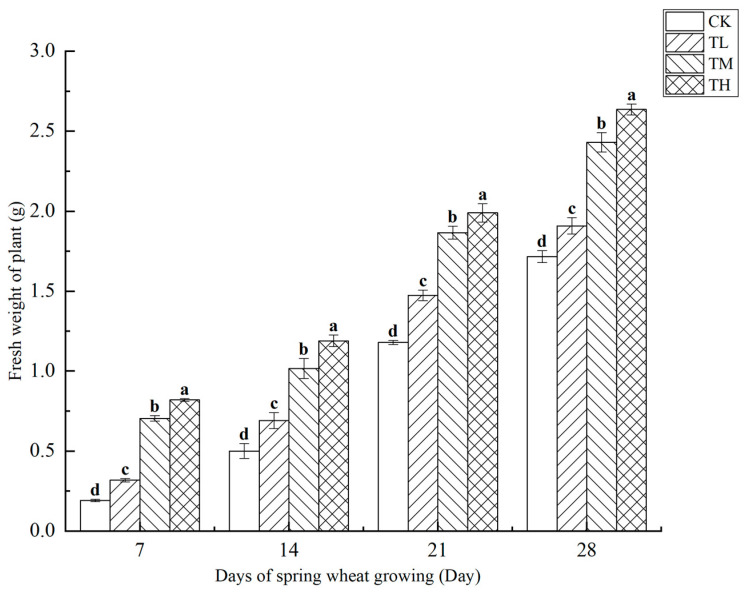
Changes in the fresh weight of plants at different stages under inoculation with different concentrations of the *B. laterosporus* YS-13 inoculant. Note: Different letters above the barsindicate significant differences (*p* < 0.05).

As shown in Figure 9, treatments with varying concentrations of *B. laterosporus* significantly increased the fresh weight of wheat plants (*p* < 0.05), with plant weight positively correlated with growth duration. Over the first 14 days, all treatment groups exhibited a significant increase in fresh weight compared to CK, with the TH, TM, and TL groups showing increases of 62.91%, 49.53%, and 21.22%, respectively (*p* < 0.05). On days 21 and 28, the fresh weight in the TL and CK groups was significantly lower than in the TH and TM groups (*p* < 0.05), with no significant difference observed between the TL and CK groups. Moreover, no significant difference was found between the TH and TM groups. By day 28, the fresh weights in the TH, TM, and TL groups had increased by 53.67%, 41.68%, and 11.23%, respectively, compared to the control (*p* < 0.05). Overall, wheat plant fresh weight increased steadily over time without any abrupt surges.

#### 3.3.3. Effect of *B. laterosporus* YS-13 at Different Concentrations on Phosphorus Content in Rootstocks

**Figure 10:**
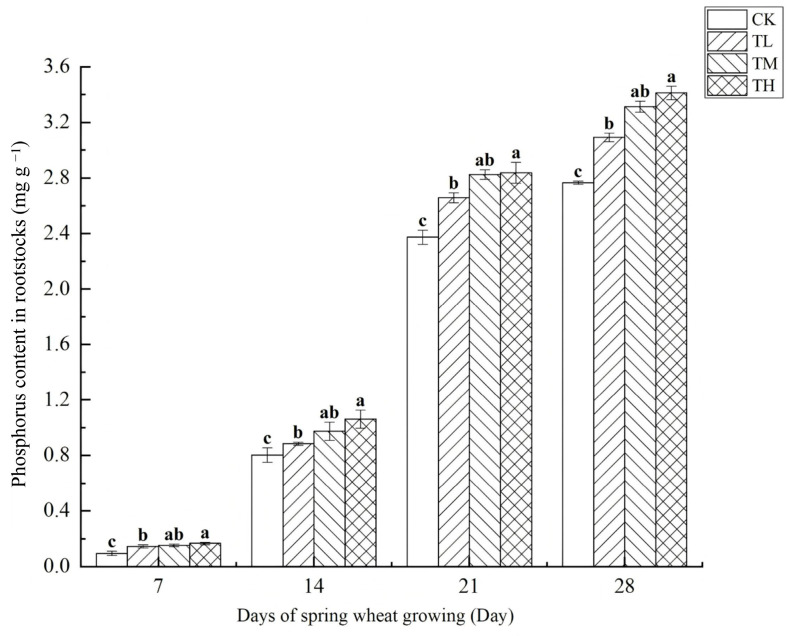
Changes in phosphorus content in rootstocks at different stages under inoculation with different concentrations of the *B. laterosporus* YS-13 inoculant. Note: Different letters above the barsindicate significant differences (*p* < 0.05).

As shown in Figure 10, treatments with varying concentrations of *B. laterosporus* significantly increased phosphorus content in rootstocks (*p* < 0.05), with a positive correlation between phosphorus content and growth duration. By day 28, the phosphorus content in the TH, TM, and TL groups had increased by 23.42%, 19.86%, and 11.78%, respectively, compared to CK, with all differences being significant (*p* < 0.05). Between days 7 and 21, phosphorus content grew rapidly, but the growth rate slowed from days 21 to 28. Overall, the TH and TM groups exhibited significantly higher phosphorus content than the TL group, indicating that inoculation with *B. laterosporus* enhanced phosphorus uptake.

#### 3.3.4. Effect of *B. laterosporus* YS-13 at Different Concentrations on the Total Root Length of Wheat

**Figure 11:**
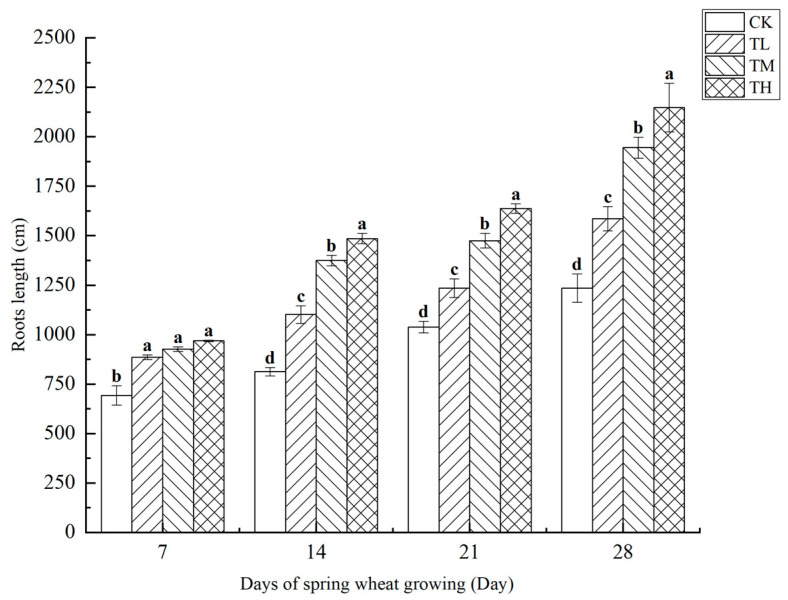
Changes in root length at different stages under inoculation with different concentrations of the *B. laterosporus* YS-13 inoculant. Note: Different letters above the barsindicate significant differences (*p* < 0.05).

As shown in Figure 11, treatments with different concentrations of *B. laterosporus* significantly increased the total root length of wheat (*p* < 0.05). By day 28, the total root length in the TH group had increased by 73.92% compared to CK, showing the most significant effect and highlighting the strong promoting effect of *B. laterosporus* YS-13 on wheat root growth. No significant differences were observed among the treatment groups on day 7. However, from day 7 onwards, significant differences emerged among the TH, TM, and TL groups (*p* < 0.05). By day 28, the total root lengths in the TH, TM, and TL groups increased by 73.92%, 57.56%, and 28.40%, respectively, compared to the control (*p* < 0.05).

#### 3.3.5. Effect of *B. laterosporus* YS-13 at Different Concentrations on the Average Diameter of Roots

**Figure 12:**
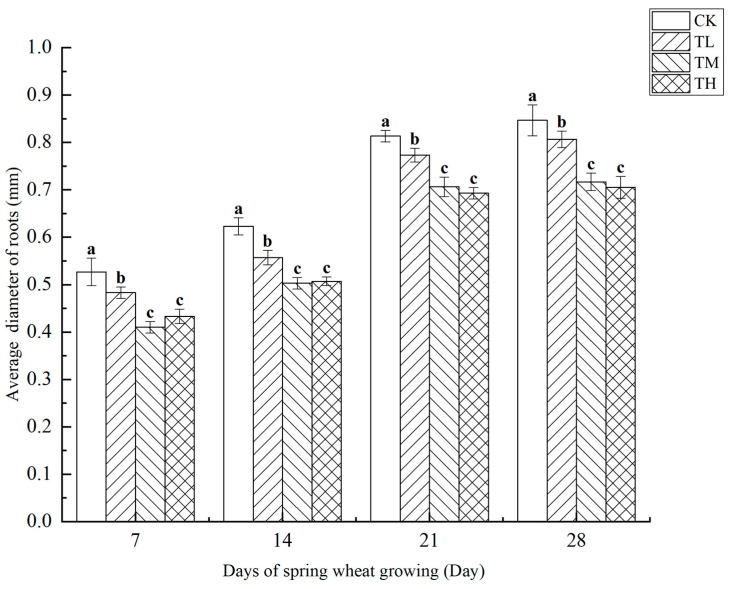
Changes in the average diameter of roots at different stages under inoculation with different concentrations of the *B. laterosporus* YS-13 inoculant. Note: Different letters above the barsindicate significant differences (*p* < 0.05).

As shown in Figure 12, the average root diameter of wheat was measured at four growth stages under treatments with different concentrations of *B. laterosporus*. The results revealed a negative correlation between root diameter and bacterial concentration, with higher inoculation concentrations leading to smaller root diameters. Throughout the 28-day growth period, significant differences in average root diameter were observed among the TH, TM, TL, and CK groups at all time points (*p* < 0.05). Notably, the TH group exhibited significant differences compared to both the TL and TM groups (*p* < 0.05), but no significant difference was found between the TH and TL groups. A sudden increase in average root diameter was observed between days 7 and 21.

#### 3.3.6. Effect of *B. laterosporus* YS-13 at Different Concentrations on the Total Root Surface Area of Roots

**Figure 13:**
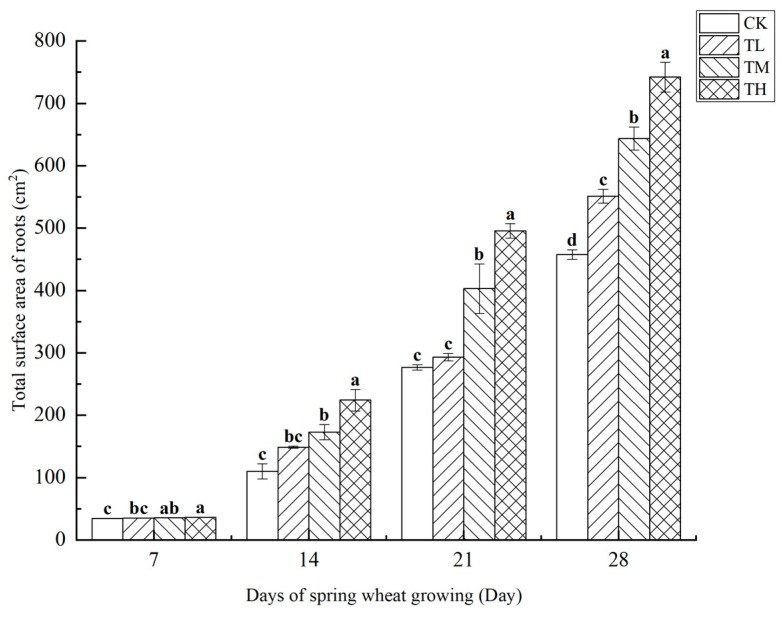
Changes in the total surface area of roots at different stages under inoculation with different concentrations of the *B. laterosporus* YS-13 inoculant. Note: Different letters above the barsindicate significant differences (*p* < 0.05).

As shown in Figure 13, treatments with different concentrations of *B. laterosporus* significantly increased the total root surface area of roots (*p* < 0.05), with a positive correlation observed between root surface area and growth duration. By day 28, significant differences in total root surface area were noted between the TH, TM, and TL groups and CK, indicating that the phosphate-solubilizing bacterium *B. laterosporus* YS-13 significantly enhanced wheat root surface area. During the first 7 days, no significant differences were observed among the TH, TM, and TL groups. However, by days 14, 21, and 28, significant differences emerged among the treatment groups (*p* < 0.05), although no significant difference was seen between the TM and TL groups at day 14. The most rapid increase in root surface area occurred between days 7 and 21, with the TH group showing the greatest increase compared to the CK group by day 28 (*p* < 0.05).

#### 3.3.7. Effect of *B. laterosporus* YS-13 at Different Concentrations on the Total Root Volume of Roots

**Figure 14:**
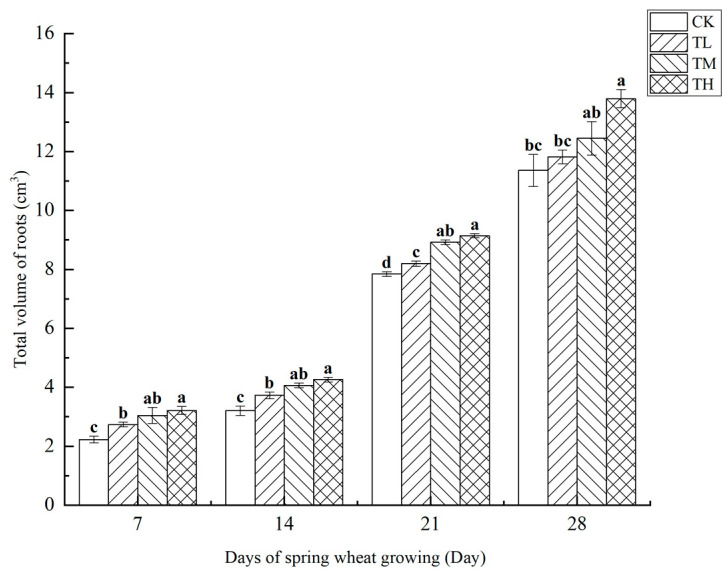
Changes in the total volume of roots at different stages under inoculation with different concentrations of the *B. laterosporus* YS-13 inoculant. Note: Different letters above the barsindicate significant differences (*p* < 0.05).

As shown in Figure 14, the total root volume of wheat treated with different concentrations of *B. laterosporus* was measured at four growth stages. The results indicated a positive correlation between root volume and growth duration. Significant differences were observed between the TH and TM groups, as well as between the TM and TL groups, at both 7 and 14 days (*p* < 0.05). On day 7, the TH group showed a significant difference compared to the TM group. By day 28, no significant difference was observed between the TH and TM groups, while the TH group remained significantly different from the TL group (*p* < 0.05). No significant differences were detected among the treatment groups on days 14 and 21. Throughout the 28-day growth period, the total root volume of wheat increased steadily without any periods of sudden or exponential growth.

## 4. Discussion

Research on PSB has largely concentrated on genera such as *Rhizobium*, *Bacillus*, *Pseudomonas*, *Serratia*, and *Enterobacter*. Among these, studies on phosphate solubilization by *Bacillus* species, particularly *Bacillus licheniformis* [42], are well-documented. While research on phosphate solubilization by *B. laterosporus* has been somewhat limited, recent studies, particularly those published in 2024, have begun to explore this topic in more depth. Recent studies have shown that *B. laterosporus* has a wide range of applications in phosphorus solubilization and as a biocontrol agent. For example, it has been reported that *B. laterosporus* enhances phosphorus utilization in plants by producing organic acids and enzymes that solubilize insoluble phosphorus sources such as calcium phosphate and iron phosphate [43]. Furthermore, its biocontrol potential has been widely proven, with multiple studies showing its significant antagonistic activity against soil pathogens such as *Fusarium* and *Pythium*, making it a promising candidate for sustainable agricultural practices [44]. Further research has demonstrated that *B. laterosporus* exhibits multiple modes of action in integrated pest management, and as a microbial pesticide, it holds great promise for commercialization in pest control [45]. However, there is limited research on phosphate solubilization by *B. laterosporus*. This study highlighted the significant degradation activity of *B. laterosporus* YS-13 against lecithin.

Phosphate solubilization is typically assessed using three methods: the solid plate method (used in this study), the molybdenum–antimony colorimetric method for measuring soluble phosphate in the supernatant, and the evaluation of available phosphorus content in soil [40,46]. In this study, lecithin was used as a phosphorus source to initially screen PSB. After isolating a strain with a high D/d ratio, further plate tests were conducted using other phosphorus sources. The results showed that *B. laterosporus* YS-13 was capable of degrading both organic and inorganic phosphorus sources, although its solubilization of inorganic phosphorus was less effective. Different phosphate-solubilizing microorganisms produce various types of organic acids depending on the phosphorus source [39,47], which may explain the strain’s preference for certain insoluble inorganic phosphorus sources. The solubilization of organic phosphorus by the strain YS-13 might occur through the secretion of phospholipases (e.g., phospholipase C/D) that break down lecithin into phosphate groups and glycerol [48,49], or through the secretion of organic acids such as citric acid and oxalic acid to chelate the released phosphate groups [50]. The solubilization of inorganic phosphorus by the strain YS-13 might occur through the secretion of organic acids (e.g., gluconic acid, lactic acid) to lower the pH and promote dissolution or possibly by producing siderophores and other chelating agents to bind calcium ions [51,52]. The solubilization of lecithin by the strain YS-13 was significantly more efficient than that of other phosphorus sources, possibly due to the strain’s special adaptation to organic phosphorus metabolism pathways or its high efficiency in recognizing and utilizing lecithin as an organic phosphorus source. Some researchers have proposed that phosphate solubilization calculations should account for bacterial phosphorus assimilation during growth, as neglecting this factor may lead to an underestimation of solubilization capacity [53]. However, this study considers assimilated phosphorus as irrelevant for practical applications and, therefore, used the molybdenum–antimony colorimetric method to measure soluble phosphorus in the supernatant. To ensure accurate results, strict control of the color development time and light exposure conditions was essential. Additionally, since soluble phosphorus content in liquid culture changes over time, the dilution factor had to be carefully adjusted to ensure that the OD_720_ values remained within the standard curve range, minimizing errors. Extensive preliminary and parallel experiments were conducted to optimize the measurement conditions and dilution factors.

Microorganisms face inherent limitations that make their direct application in practical production challenging. As a result, current approaches typically focus on optimizing fermentation processes to produce bioactive substances beneficial to plants [54]. This optimization generally involves two key aspects: improving fermentation media and refining fermentation conditions [55]. Common optimization methods include single-factor experiments, orthogonal experiments, central composite design (CCD), and response surface methodology (RSM). Among these, RSM is especially effective for analyzing the interactions between multiple factors and provides more reliable data for optimizing fermentation conditions [56]. RSM also enables the creation of regression models to generate three-dimensional response surfaces and contour plots, visually representing the impact of various factors on target variables [57]. In this study, a combined approach of single-factor experiments and RSM was used to optimize the fermentation conditions for *B. laterosporus* YS-13, with viable cell count as the evaluation metric. The growth curve and optimal growth conditions were first established, followed by single-factor experiments to identify the best carbon source, nitrogen source, and initial pH. RSM was then applied to determine the optimal fermentation medium formulation.

Phosphorus is an essential nutrient for wheat growth and development. It is a key component of various organic compounds and plays a vital role in numerous metabolic processes. Soil is the primary source of phosphorus for wheat, but much of it is bound in insoluble minerals, making it less available for plant uptake and reducing its utilization efficiency, which negatively affects wheat growth and yield [58]. To enhance wheat yield, soluble phosphorus fertilizers are commonly used. However, these fertilizers often become quickly fixed in the soil, transforming into forms that are difficult for wheat to absorb, with utilization rates generally below 25% [59]. As a result, large amounts of insoluble phosphorus accumulate in the soil, particularly in the calcareous soils of northern China, where this fixed phosphorus constitutes a significant portion. Releasing this bound phosphorus is crucial to improving soil phosphorus availability and reducing the reliance on phosphorus fertilizers.

PSB are considered the most effective strain for releasing mineral phosphorus. However, their functionality is often limited by their low abundance in the wheat rhizosphere [60,61]. To overcome this limitation, it is essential to screen phosphate-solubilizing strains under natural conditions, develop microbial inoculants, and apply them to wheat-growing soils to enhance wheat growth [62]. Although the optimal fermentation conditions of *B. laterosporus* YS-13 were determined under controlled laboratory settings (optimal pH 7.6), its field application involves more complex soil environments. The soil used in this study had a pH of 7.35, slightly above the optimal range for wheat growth (commonly pH 6.0–7.5). Nevertheless, *B. laterosporus*, as a spore-forming bacterium, is known for its high environmental tolerance and has been reported to maintain viability and functional activity across a wide pH range (5.5–8.0) [45,63]. It has also demonstrated plant growth-promoting effects under various pH conditions [64].

The present study focused on the development of a stable microbial inoculant rather than simulating field conditions during fermentation. The optimization of viable cell production aims to ensure efficacy upon application. While soil conditions may affect microbial proliferation, the environmental adaptability of *B. laterosporus* suggests its potential effectiveness in diverse agricultural settings. Further studies will address field-scale validation and provide guidelines for optimal application conditions, including suitable soil pH ranges. Furthermore, this strain may have contributed to the enhancement of the soil microbial community, promoting a more favorable environment for nutrient uptake and plant health. These findings suggest that *B. laterosporus* YS-13 holds significant potential as a microbial inoculant for sustainable agricultural practices, particularly in phosphorus-deficient soils, where it could aid in optimizing crop productivity while reducing the reliance on chemical fertilizers.

## 5. Conclusions

Through this study, we isolated a total of 54 bacterial strains, 7 actinobacteria strains, and 3 fungal strains from soil samples and found a significant relationship between the phosphate-solubilizing capacity and plant growth promotion of *B. laterosporus* YS-13, indicating that this strain not only efficiently solubilizes various forms of organic and inorganic phosphorus but also significantly promotes wheat growth. These findings have important implications for the development of efficient biofertilizers and the enhancement of crop phosphorus utilization. Specifically, strain YS-13 exhibited high viable cell counts under optimized fermentation conditions and significantly improved plant growth parameters and phosphorus absorption in pot experiments, demonstrating its potential for sustainable agricultural development. Although this study reveals the key role of strain YS-13 in promoting crop growth and phosphorus utilization, its performance across different crops, soil types, and real field conditions still needs further validation. Future research could deepen the understanding of this strain’s agricultural potential by expanding field trials, exploring its interactions with soil microbial communities, and developing efficient formulations.

## Figures and Tables

**Table 1:** The phosphorus-solubilizing characteristics of the strain YS-13 with different phosphorus sources.

Strain YS-13	D (mm)	d (mm)	D/d	Available Phosphorus (mg L^−1^)
Calcium phytate	18.59 ± 0.44 b	8.89 ± 0.68 b	2.09	17.05 ± 3.43 b
Lecithin	23.34 ± 2.20 a	9.97 ± 0.88 a	2.34	21.24 ± 4.71 a
Calcium phosphate	11.33 ± 0.27 c	6.59 ± 0.65 c	1.63	11.73 ± 2.26 c
Iron phosphate	11.17 ± 1.32 c	9.31 ± 0.70 ab	1.20	6.67 ± 2.09 d

Note: Different letters above the barsindicate significant differences (*p* < 0.05).

**Table 2:** Morphological and Gram staining characteristics of the strain YS-13.

Strain YS-13	Characteristics
Shape and surface characteristics	Circular or oval, with a raised center
Transparency and glossiness	Non-glossy and semi-transparent
Edge and color	Smooth and regular edges, yellow-brown in color
Cell morphology	Short and thick rod-shaped, with blunt and rounded ends
Gram staining	Gram-positive during the logarithmic phase; Gram-negative during the late stationary phase and the decline phase
Spore morphology and location	Oval-shaped, subterminally located

**Table 3:** Physiological and biochemical characteristics of the strain YS-13.

Indicator		Strain YS-13
Catalase reaction		+
Strictly aerobic		−
Methyl red reaction		+
Voges–Proskauer (V-P) reaction		−
Nitrate reduction		+
Indole production		−
Citrate utilization		−
Starch hydrolysis		+
Casein hydrolysis		+
H_2_S		−
Sugar alcohol fermentation	Glucose	+
	Lactose	−
	Mannitol	+
	Gas production from glucose	−
Salt tolerance	2% NaCl	+
	5% NaCl	−
	7% NaCl	−
Growth temperature	45 °C	+
	65 °C	−

Note: + indicates a positive reaction; − indicates a negative reaction.

**Table 4:** Response surface test factors and levels.

Source	Fermentation Conditions	Level	
		−1	+1
*X*_1_	Glucose concentration (g L^−1^)	4	10
*X*_2_	Peptone concentration (g L^−1^)	4	10
*X*_3_	Sodium chloride concentration (g L^−1^)	0.1	0.5
*X*_4_	Temperature (°C)	30	36
*X*_5_	Initial pH	6	8
*X*_6_	Amount of inoculum (%)	4	8
*X*_7_	Agitation speed (r min^−1^)	1.5	4.5
*X*_8_	Cultivation time (h)	24	48

**Table 5:** Design matrix and experimental results of the Plackett–Burman design.

Assay	Variable Levels								Number of Live Bacteria (×10^8^ CFU mL^−1^)
	*X*_1_	*X*_2_	*X*_3_	*X*_4_	*X*_5_	*X*_6_	*X*_7_	*X*_8_	
1	1	1	1	−1	−1	−1	1	1	4.2
2	−1	−1	−1	−1	−1	1	−1	−1	3.1
3	1	−1	−1	−1	−1	1	−1	1	4.24
4	1	−1	1	1	−1	1	1	−1	3.44
5	1	−1	−1	−1	1	−1	1	−1	3.67
6	−1	1	1	−1	1	1	1	−1	3.7
7	1	−1	1	1	1	−1	−1	1	3.84
8	−1	1	1	1	−1	−1	−1	−1	3.47
9	1	1	−1	1	1	1	−1	−1	4.69
10	−1	−1	1	−1	1	1	−1	1	3.4
11	−1	1	−1	1	1	−1	1	1	4.08
12	−1	−1	−1	1	−1	1	1	1	3.2

**Table 6:** Results of the climbing experiment.

Assay	*X*_1_ (g L^−1^)	*X*_2_ (g L^−1^)	*X*_5_	Number of Live Bacteria (×10^8^ CFU mL^−1^)
1	4	4	6	3.37
2	5.5	5.5	6.5	4.12
3	7	7	7	5.54
4	8.5	8.5	7.5	6.11
5	10	10	8	5.84

**Table 7:** Design and results of the response surface.

Assay	Variable Levels			Number of Live Bacteria (×10^8^ CFU mL^−1^)
	*X*_1_	*X*_2_	*X*_5_	
1	0	0	0	6.35
2	1	0	–1	5.72
3	0	0	0	6.24
4	–1	0	1	5.43
5	1	1	0	5.88
6	–1	0	–1	5.11
7	0	1	–1	5.65
8	0	0	0	6.39
9	–1	–1	0	4.93
10	0	1	1	5.82
11	–1	1	0	5.74
12	1	–1	0	5.78
13	0	0	0	6.36
14	0	–1	1	5.34
15	0	–1	–1	5.02
16	0	0	0	6.3
17	1	0	1	5.86

## Data Availability

The original contributions presented in the study are included in the article; further inquiries can be directed to the corresponding authors.

## Appendix A

**Table A1:** Significance analysis of the Plackett–Burman experiments.

Source	Sum of Squares	df	Mean Square	*F*	*p*
Model	2.35	8	0.2935	15.87	0.0221 *
*X*_1_-Glucose	0.8533	1	0.8533	46.13	0.0065 **
*X*_2_-Peptone	1.04	1	1.04	56.45	0.0049 **
*X*_3_-Sodium chloride	0.1045	1	0.1045	5.65	0.0979
*X*_4_-Temperature	0.0096	1	0.0096	0.5207	0.5227
*X*_5_-Initial pH	0.2700	1	0.2700	14.59	0.0316 *
*X*_6_-Amount of inoculum	0.0048	1	0.0048	0.2595	0.6456
*X*_7_-Agitation speed	0.0056	1	0.0056	0.3045	0.6195
*X*_8_-Cultivation time	0.0560	1	0.0560	3.03	0.1802

Note: * Indicates significant difference, *p* < 0.05; ** Indicates extremely significant difference, *p* < 0.01.

**Table A2:** Response surface ANOVA.

Source	Sum of Squares	df	Mean Square	*F*	*p*
model	3.59	9	0.3994	144.82	<0.0001 **
*X*_1_	0.5151	1	0.5151	186.78	<0.0001 **
*X*_2_	0.5101	1	0.5101	184.94	<0.0001 **
*X*_5_	0.1128	1	0.1128	40.91	0.0004 **
*X*_1_ *X*_2_	0.1260	1	0.1260	45.70	0.0003 **
*X*_1_ *X*_5_	0.0081	1	0.0081	2.94	0.1303
*X*_2_ *X*_5_	0.0056	1	0.0056	2.04	0.1963
*X*_1_ *^2^*	0.4768	1	0.4768	172.88	<0.0001 **
*X*_2_ *^2^*	0.7043	1	0.7043	255.39	<0.0001 **
*X* ^2^	0.8968	1	0.8968	325.17	<0.0001 **
Residua	0.0193	7	0.0028		
Lack of Fit	0.0054	3	0.0018	0.5211	0.6904
Pure Error	0.0139	4	0.0035		
Cor Total	3.61	16			

Note: ** Indicates extremely significant difference, *p* < 0.01.
